# Glaucoma Is Associated with the Risk of Obstructive Sleep Apnea: A Population-Based Nationwide Cohort Study

**DOI:** 10.3390/diagnostics12122992

**Published:** 2022-11-29

**Authors:** So Yeon Lee, Hyunjae Yu, Dong-Kyu Kim

**Affiliations:** 1 Department of Ophthalmology, Nune Eye Hospital, Seoul 06198, Republic of Korea; 2 Institute of New Frontier Research, Division of Big Data and Artificial Intelligence, Chuncheon Sacred Heart Hospital, Hallym University College of Medicine, Chuncheon 24253, Republic of Korea; 3 Department of Otorhinolaryngology-Head and Neck Surgery, Chuncheon Sacred Heart Hospital, Hallym University College of Medicine, Chuncheon 24253, Republic of Korea

**Keywords:** glaucoma, obstructive sleep apnea, cohort, risk

## Abstract

The association between glaucoma and the risk of obstructive sleep apnea (OSA) has not been fully evaluated. Therefore, this study aimed to investigate the prospective association between glaucoma and OSA. In total, 1437 patients with glaucoma and 5748 patients without glaucoma were enrolled after 1:4 propensity score matching using a nationwide cohort sample. We investigated OSA events during a 10-year follow-up period. Survival analysis, the log-rank test, and Cox proportional hazards regression models were used to calculate the incidence, disease-free survival rate, and hazard ratio (HR). The incidence of OSA was 12,509.0 person-years among those with glaucoma. The adjusted HR for patients with glaucoma developing OSA events during the follow-up period was 1.52 (95% confidence interval [CI]: 0.64–3.621) after other covariates. In a subgroup analysis, primary angle-closure glaucoma (PACG) showed a significantly increased adjusted HR for OSA events (5.65, 95% CI: 1.65–19.41), whereas we could not find any significant association between primary open-angle glaucoma (POAG) and OSA. The adjusted HR of OSA events in POAG was considerably increased 4 years after POAG diagnosis. PACG may be associated with an increased incidence of OSA. Clinicians should pay attention to early detection of OSA in patients with PACG.

## 1. Introduction

Glaucoma is a group of eye disorders, which are progressive, chronic optic neuropathies resulting from the degeneration of retinal ganglion cells, leading to visual impairment. It is the most common form of optic nerve damage that leads to vision loss if left untreated [1]. Although early detection and management could decrease the physical and economic burden, early stage glaucoma is usually asymptomatic; thus, it often remains undetected until the late stage of the disease [2,3]. Glaucoma is usually divided into two subtypes based on the mechanisms of optic nerve damage: normal-tension glaucoma (NTG), primary angle-closure glaucoma (PACG), and primary open-angle glaucoma (POAG). The prevalence of PACG is highest in Asia, and PACG is more likely to result in blindness than POAG [4,5,6,7]. The exact mechanism of racial differences in risk and the potential mechanistic pathways remain unclear.

Obstructive sleep apnea (OSA) is characterized by repeated cessation of breathing during sleep and is primarily caused by complete or partial airway obstruction. These episodes of airway obstruction induce nocturnal hypoxemia, hypercapnia, and sleep fragmentation. OSA can also cause or exacerbate severe, major organ disorders, including cardiovascular disease, metabolic syndrome, and neurocognitive deterioration [8,9,10,11]. For these reasons, several studies also suggested that, in ocular tissues, OSA could contribute to reducing the ocular perfusion pressure and decrease oxygenation to the optic nerve, which eventually leads to glaucomatous optic neuropathy [12,13,14,15]. In addition, most previous studies have described that a change in the intraocular pressure during apnea in patients with OSA is a major risk factor for glaucomatous changes in patients with OSA. However, few studies have investigated the risk of OSA in patients with glaucoma. Therefore, to further investigate the relationship between these two diseases, we examined the association between glaucoma and the prospective risk of OSA using a representative sample from the National Sample Cohort data.

## 2. Materials and Methods

### 2.1. Ethics Statements

This study was approved by the Institutional Review Board (IRB) of Hallym Medical University Chuncheon Sacred Hospital (IRB number: 2016-05-052), and the requirement for written informed consent was waived by the IRB because the South Korea National Health Insurance Service (KNHIS)–National Sample Cohort database used in the study comprised de-identified secondary data.

### 2.2. Study Design and Participants

This study was designed as a retrospective, nationwide, propensity score-matched cohort study using a dataset from the national health claims database. We used a representative sample of 1,025,340 adults from the 2002–2013 KNHIS–National Sample Cohort in South Korea. This dataset accounts for approximately 2.2% of the South Korean population in 2002. Stratified random sampling was performed using 1476 strata by age (18 groups), sex (2 groups), and income level (41 groups: 40 health insurance groups and 1 medical aid beneficiary) among the South Korean population of 46 million in 2002. Additionally, the KNHIS–National Sample Cohort contains data from all health services, including hospital visits (inpatient and outpatient), medical procedures, drug prescriptions, hospital diagnoses, and demographic information (including sex, age, household income, and mortality) during the study period (2002–2013).

**Figure 1:**
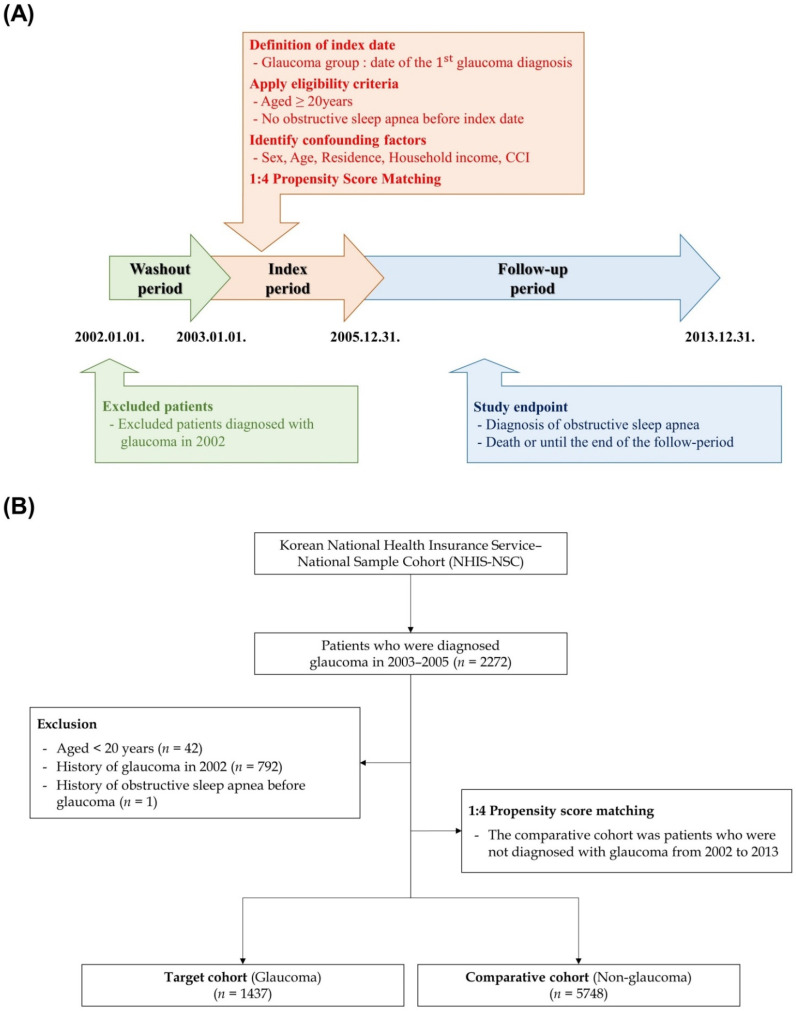
(**A**) Schematic description of study design. (**B**) Flow diagram of the enrolled patients. CCI, Charlson comorbidity index.

All disease diagnostic codes were identified using the International Classification of Diseases, Tenth revision. The primary glaucoma group (H40.1 and H40.2) included all patients who received inpatient or outpatient care for an initial diagnosis of glaucoma during the index period (January 2003 and December 2005). To remove any potential preexisting cases of OSA, we established a washout period in the first year (2002). Additionally, we excluded (1) patients aged <20 years, (2) patients who died during the index period, (3) patients diagnosed with OSA before the diagnosis of primary glaucoma. Next, we selected the comparison group (non-glaucoma) using propensity score-matching methodology and randomly identified propensity score-matched participants from the remaining cohort registered in the database as 4 participants without glaucoma for each patient with glaucoma. The operational definitions of the study endpoints were all-cause mortality and the incidence of OSA (G47.3). If patients showed no events or were still alive until 31 December 2013, they were censored after this time point. Finally, 1437 eligible patients with glaucoma and 5748 patients in the comparison group were enrolled in this study. In brief, we present a schematic of the study design and flow of the study setting in Figure 1.

### 2.3. Predictor and Outcome Variables

The study population was divided into 3 age groups (<45, 45–64, >64 years), 3 income groups (low: ≤30%, middle: 30.1–69.9%, and high: ≥70% of the median), 3 residential areas (first area: Seoul, the largest metropolitan region in South Korea; second area: other metropolitan cities in South Korea; and third area: small cities and rural areas), and the Charlson comorbidity index (CCI) (score: 0, 1, and 2). The risks of OSA in the glaucoma and comparison groups were compared using person-years at risk, which were defined as the duration between the date of glaucoma diagnosis or 1 January 2003 (for the comparison group) and the patient’s respective endpoint. Comorbidity was adjusted using the CCI, which is a weighted index to predict the risk of death within 1 year of hospitalization for patients with specific comorbid conditions. It was determined based on patient medical records and converted into ICD-10 codes for 19 diseases to be used as administrative data.

### 2.4. Statistical Analysis

Incidence rates per 1000 person-years for OSA were obtained by dividing the number of patients with incidents of specific diseases by the person-years at risk. To identify whether glaucoma increased the risk of occurrence of specific diseases, we used Cox proportional hazard regression analyses to calculate the hazard ratios (HRs) and 95% confidence intervals (CIs) adjusted for the other predictor variables. During the follow-up period, the Kaplan–Meier method was used to calculate OSA-free survival among patients with glaucoma. All statistical analyses were performed using R, version 4.0.0 software with a significance level of a 2-tailed *p*-value of 0.05.

## 3. Results

### 3.1. Demographic and Clinical Characteristics

The present study comprised 1437 patients with glaucoma and 5748 individuals without glaucoma (comparison) during a 10-year follow-up period. Table 1 presents patient characteristics, including sex, age, residence, household income, disability, and comorbidities. The distributions of sex, age, residential area, household income, and comorbidities were similar between the groups. This finding means that these variables were appropriately matched, and we confirmed whether the matching was performed appropriately using the balance plot technique (Figure S1). We also used univariate and multiple Cox regression models to analyze the HRs for OSA development during the 10-year follow-up period. The data for time to event and the censored event are shown in Table 2.

### 3.2. Effect of Glaucoma on the Risk of Subsequent Development of Obstructive Sleep Apnea

**Figure 2:**
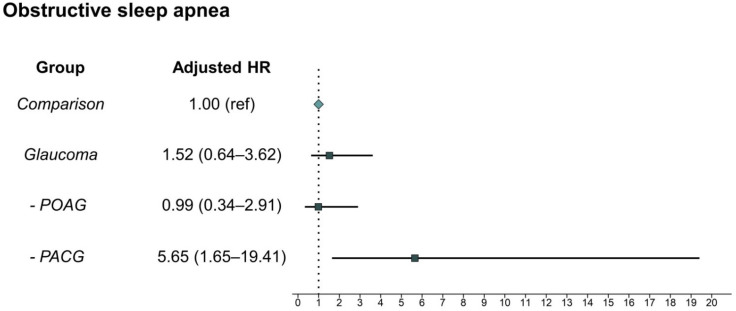
Comparison of the risk for obstructive sleep apnea development between the comparison group and glaucoma, POAG, and PACG groups. POAG, primary open-angle glaucoma; PACG, primary angle-closure glaucoma; HR, hazard ratio; Comparison, individuals without glaucoma.

**Figure 3:**
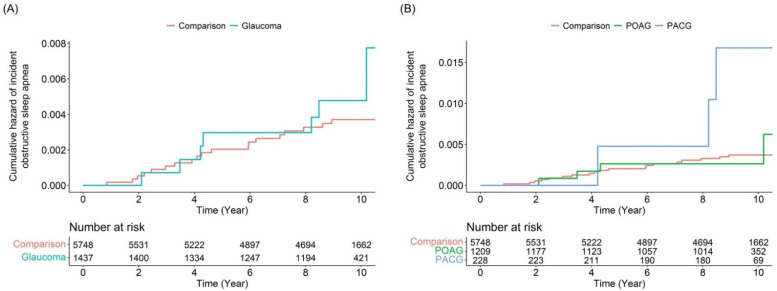
Kaplan–Meier survival curves and log-rank test results for the development of incident obstructive sleep apnea: patients with (**A**) glaucoma and those with (**B**) POAG and PACG. POAG, primary open-angle glaucoma; PACG, primary angle-closure glaucoma; Comparison, individuals without glaucoma.

The incidences of OSA were 0.56 per 1000 person-years in the glaucoma group and 0.37 per 1000 person-years in the comparison group (Table 3). Thus, the incidence of OSA in the glaucoma group was nearly one-and-a-half as high as that in the non-glaucoma group. However, there was no significant difference in the subsequent development of OSA between the groups. Interestingly, subgroup analysis revealed that patients with PACG showed an increased risk of OSA events (adjusted HR: 5.65, 95% CI: 1.65–19.41), unlike patients with POAG (adjusted HR: 0.99, 95% CI: 0.34–2.90). The risk of subsequent development of OSA according to glaucoma subtype is presented in Figure 2. Kaplan–Meier survival curves with log-rank test results indicated that patients with PACG developed OSA events more frequently than individuals without glaucoma (Figure 3). Yet, the overall glaucoma or POAG groups had no significant specific disease (OSA)-free survival rate compared with the comparison group. In the analysis of HRs over time, the risk of OSA development in patients with PACG increased in a time-dependent manner (Table 4). Specifically, we detected a low risk ratio for developing OSA after PACG diagnosis within the first 4 years. Additionally, the adjusted HR of OSA events dramatically increased after 8 years after the diagnosis of PACG.

Furthermore, we performed an analysis of the risk for newly developing OSA according to the comorbidities. It revealed that patients with PACG who had fewer comorbidities showed a higher risk of incident OSA events (Table 5).

## 4. Discussion

To our best knowledge, this longitudinal study is the first to examine the association between glaucoma and the prospective risk of OSA using a nationwide representative sample dataset. We found that patients with PACG were associated with a higher risk of developing OSA than those without PACG; however, we observed no overall association between glaucoma and an increased incidence of OSA. Specifically, our findings revealed that the risk of OSA development is associated with the duration of PACG diagnosis. Therefore, in the present study, we detected novel findings between the two diseases; however, we still could not exactly determine these findings whether meaningful associations or only temporal incidental findings.

Previous cross-sectional studies have reported that a change in the intraocular pressure during apnea contributes to the development of glaucomatous changes in patients with OSA [14,16,17,18]. One study on NTG reported that the proportion of patients with moderate-to-severe OSA were significantly higher among patients with NTG than in those without NTG [16]. Other studies have also demonstrated that patients with severe OSA have a significantly thinner retinal nerve fiber layer (RNFL) than those with normal to mild OSA [17,18]. Additionally, one study showed the thinning of the RNFL superotemporally by 1.5 µm with an increase in the apnea-hypopnea index by 5 events per hour [19]. Several studies have described the occurrence of OSA in patients with glaucoma [12,20,21,22]. One study demonstrated that patients with severe OSA had >8 times RNFL thickness progression than patients with none or mild OSA [20]. Another study showed a higher incidence of OSA in patients with POAG [12]. Similarly, in this study, we found an almost five-fold risk ratio for OSA development in patients with PACG. However, unlike those aforementioned studies, some other studies have suggested that sleep apnea is not a risk factor for glaucoma progression in patients with OSA [23,24]. Consistent with these findings, our study showed no significant increase in the risk of OSA in patients with POAG. Additionally, we found that the adjusted HR of OSA events was higher in having a fewer comorbidities group than in having a more comorbidities group. It means that various comorbidities could influence the development of OSA; thus, the difference in the risk for OSA events was little between non-glaucoma and PACG groups, specifically in participants having more comorbidities.

Generally, POAG is characterized by obstruction of the aqueous humor pathway because of trabecular meshwork degeneration. This obstruction prevents the exit of the aqueous humor, resulting in increased intraocular pressure, which is often thought to damage the optic nerve. Angle closure is a fundamental pathological mechanism of PACG. Thus, high intraocular pressure is secondarily induced due to angle closure. A shallow anterior chamber, thickened lens, hyperopic refractive error, and short axial length are also commonly observed in patients with PACG [25,26,27]. Among these, the most important anatomical risk factors for PACG are shallowness and a narrow angle of the anterior chamber [28]. Overall, PACG is less common than POAG, but both diseases showed different incidence rates according to race [28,29,30]. PACG is more prevalent in Chinese people, Asian Indians, and Eskimos. To date, various studies have demonstrated a genetic link to the development of PACG in these populations, although a clear genetic involvement remains unclear [31,32,33]. Recently, several studies have suggested that susceptible single nucleotide polymorphisms (SNPs) in the matrix metalloproteinase-9 (*MMP-9*) gene are associated with PACG development. The *MMP-9* gene encodes a family of zinc- and calcium-dependent enzymes with proteolytic activity that is involved in remodeling of the extracellular matrix [34,35,36]. One study demonstrated that the SNP located in *MMP-9* might be associated with PACG in the Southern Chinese population [34]. Another study reported that a different SNP in *MMP-9* is associated with susceptibility to acute PACG in Taiwanese patients [35]. Additionally, a previous study showed an association between the SNP in the *MMP-9* gene and PACG in an Australian population [36]. Interestingly, *MMP-9* expression is also a risk factor for cardiovascular diseases in patients with OSA. It recruits neutrophils and monocytes into the subendothelial layer, which induces the formation of foam cells, which is considered one of the major processes of atherosclerosis. Although there is no distinct evidence for the link between PACG and OSA, we believe that genetic variation, such as the *MMP-9* gene, may contribute to this. Furthermore, other studies showed that some patients with glaucoma had the SNP in the promoter of apolipoprotein E (APOE) [37,38]. APOE is also known as the association of an increased risk of neurocognitive dysfunction in OSA patients [39].

Although we could not present the exact mechanism between PACG and OSA, our study has several unique advantages. First, we used a nationwide population-based dataset, which enabled us to effectively analyze all events associated with glaucoma and OSA. Second, this cohort had a long follow-up period and was adjusted for most major comorbidities using the CCI. The CCI is a method of categorizing patient comorbidities based on the ICD diagnosis codes found in the KNHIS–NSC database. Third, to improve diagnostic accuracy, we selected patients with glaucoma diagnosed by ophthalmologists, and patients with OSA were defined as those with diagnostic codes for polysomnography. Fourth, the reliability of the KNHIS-NSC database has been validated, which showed a similar prevalence of 20 major diseases for each of the years assessed [40]. Additionally, several retrospective cohort studies already published a similar study design using this database [41,42,43,44,45]. Nevertheless, the present study has some notable limitations. First, this dataset could not include other specific health data, including the body mass index, lipid profiles, and information regarding behavioral risk factors, such as smoking or alcohol consumption. Therefore, these possible confounding factors could not be controlled for in this study. Moreover, this database does not provide information from medical charts, visual field results, optical coherence tomography, or polysomnography. Therefore, we could not determine the severity of glaucoma or OSA. Furthermore, this dataset does not include the data for blood pressure (either systolic, mean, 24 h) of individual participants. However, to overcome this issue, we adjusted comorbidity including HTN as the CCI score. Thus, both groups are well-matched in HTN status (presence or absence). Second, we could not differentiate between acute and chronic forms of PACG. Unlike acute PACG, chronic PACG is a gradual, often clinically silent, closure of the angle resulting in increased intraocular pressure and eventual glaucomatous optic nerve damage. Thus, the natural course of the chronic form is often similar to that of POAG. Third, the cholinergic agonist is the first-line drug for treating PACG; however, when we calculated the risk of OSA events, we could not consider the therapeutic effect of cholinergic agonists on patients with PACG because this variable is too heterogeneous to adjust for. Finally, this was a retrospective cohort study, and we could not directly examine and analyze the mechanisms underlying the relationship between PACG and OSA. Further clinical and experimental studies are needed to confirm the possible link between these two diseases.

## 5. Conclusions

The present study examined the association between glaucoma and the risk of OSA after adjusting for clinical and demographic factors. We identified an increased risk of OSA events in patients with PACG; however, no significant association was observed between POAG and OSA. In this study, we could not conclude whether this association is the possible link between two disease or the temporal incidental finding. However, this nationwide population-based dataset allowed us to trace the entire medical service use history of >1 million South Koreans and provided a unique opportunity to examine the association between glaucoma and the risk of OSA, while adjusting for clinical and demographic factors. Therefore, we recommend that clinicians would be aware of the potential development of OSA in patients with PACG and recommend polysomnography to ensure early detection if patients with PACG have specific sleep-disordered symptoms.

## Figures and Tables

**Table 1:** Characteristics of the enrolled participants in this study.

Variable	Comparison (*n* = 5748)	Glaucoma (*n* = 1437)	*p*-Value
Sex			1.000
Male	2876 (50.0%)	719 (50.0%)	
Female	2872 (50.0%)	718 (50.0%)	
Age (years)			1.000
<45	1232 (21.4%)	308 (21.4%)	
45–64	2380 (41.4%)	595 (41.4%)	
>64	2136 (37.2%)	534 (37.2%)	
Residence			1.000
Seoul	1272 (22.1%)	318 (22.1%)	
Second area	1460 (25.4%)	365 (25.4%)	
Third area	3016 (52.5%)	754 (52.5%)	
Household income			1.000
Low (0–30%)	1064 (18.5%)	266 (18.5%)	
Middle (30–70%)	1920 (33.4%)	480 (33.4%)	
High (70–100%)	2764 (48.1%)	691 (48.1%)	
CCI			1.000
0	3100 (53.9%)	775 (53.9%)	
1	1244 (21.6%)	311 (21.6%)	
≥2	1404 (24.4%)	351 (24.4%)	

Comparison, individuals without glaucoma; Seoul, the largest metropolitan area; second area, other metropolitan cities; third area, other areas; CCI, Charlson comorbidity index.

**Table 2:** Data for time to event or censored event in this study.

		Number of Endpoint Events
Event		26
Comparison	19	
Glaucoma	7	
Total censored (No event)		7159
Comparison	5729	
Glaucoma	1430	
Termination of study		5670
Comparison	4517	
Glaucoma	1153	
Loss to follow-up/Drop-out		1489
Comparison	1212	
Glaucoma	277	

Comparison, individuals without glaucoma.

**Table 3:** Incidence per 1000 person-years and the risk of obstructive sleep apnea in patients with glaucoma.

Variable	N	Case	Person-Year	Incidence	Unadjusted HR (95% CI)	Adjusted HR (95% CI)
Group						
Comparison	5748	19	51,545.6	0.37	1.00 (ref)	1.00 (ref)
Glaucoma	1437	7	12,509.0	0.56	1.50 (0.63–3.57)	1.52 (0.64–3.62)
POAG	1209	4	10,550.2	0.38	1.01 (0.34–2.98)	0.99 (0.34–2.90)
PACG	228	3	1958.8	1.53	4.10 (1.21–13.87) *	5.65 (1.65–19.41) **

POAG, primary open-angle glaucoma; PACG, primary angle-closure glaucoma; HR, hazard ratio; CI, confidence interval; ref, reference; Comparison, individuals without glaucoma. * *p* < 0.05 and ** *p* < 0.01.

**Table 4:** Hazard ratios for incident obstructive sleep apnea associated with PACG over time.

Follow-Up Period (Year)	Number of Participants with Obstructive Sleep Apnea		Adjusted Hazard Ratio (95% Confidence Interval)
	Comparison	PACG	
1	1	0	0.00 (0–Inf)
2	3	0	0.00 (0–Inf)
3	6	0	0.00 (0–Inf)
4	8	0	0.00 (0–Inf)
5	11	1	3.09 (0.39–24.38)
6	13	1	2.46 (0.32–19.15)
7	14	1	2.32 (0.30–17.91)
8	17	1	2.02 (0.27–15.34)
9	19	3	5.65 (1.65–19.41) **
10	19	3	5.65 (1.65–19.41) **
11	19	3	5.65 (1.65–19.41) **

PACG, primary angle-closure glaucoma; Inf, infinity; Comparison, individuals without glaucoma. ** *p* < 0.01.

**Table 5:** Hazard ratios of obstructive sleep apnea by CCI between comparison (non-glaucoma) and PACG groups.

CCI	<2		≥2	
	Comparison	PACG	Comparison	PACG
Obstructive sleep apnea				
Unadjusted HR (95% CI)	1.00 (ref)	4.98 (1.09–22.73) *	1.00 (ref)	3.29 (0.42–25.99)
Adjusted HR (95% CI)	1.00 (ref)	6.98 (1.47–33.16) *	1.00 (ref)	4.76 (0.59–38.71)

CCI, Charlson comorbidity index; PACG, primary angle-closure glaucoma; HR, hazard ratio; CI, confidence interval. * *p* < 0.05.

## Data Availability

The authors confirm that the data supporting the findings of this study are available within the article.

## Supplementary Materials

The following supporting information can be downloaded at: https://www.mdpi.com/article/10.3390/diagnostics12122992/s1, Figure S1: Balance plot for 5 variables before and after matching.
