# Supplementary figures and images for: Glaucoma Is Associated with the Risk of Obstructive Sleep Apnea: A Population-Based Nationwide Cohort Study
Source: Diagnostics (Basel). 2022 Nov 29;12(12):2992. doi: 10.3390/diagnostics12122992 (PMC9776797; doi:10.3390/diagnostics12122992)

Distributional Balance

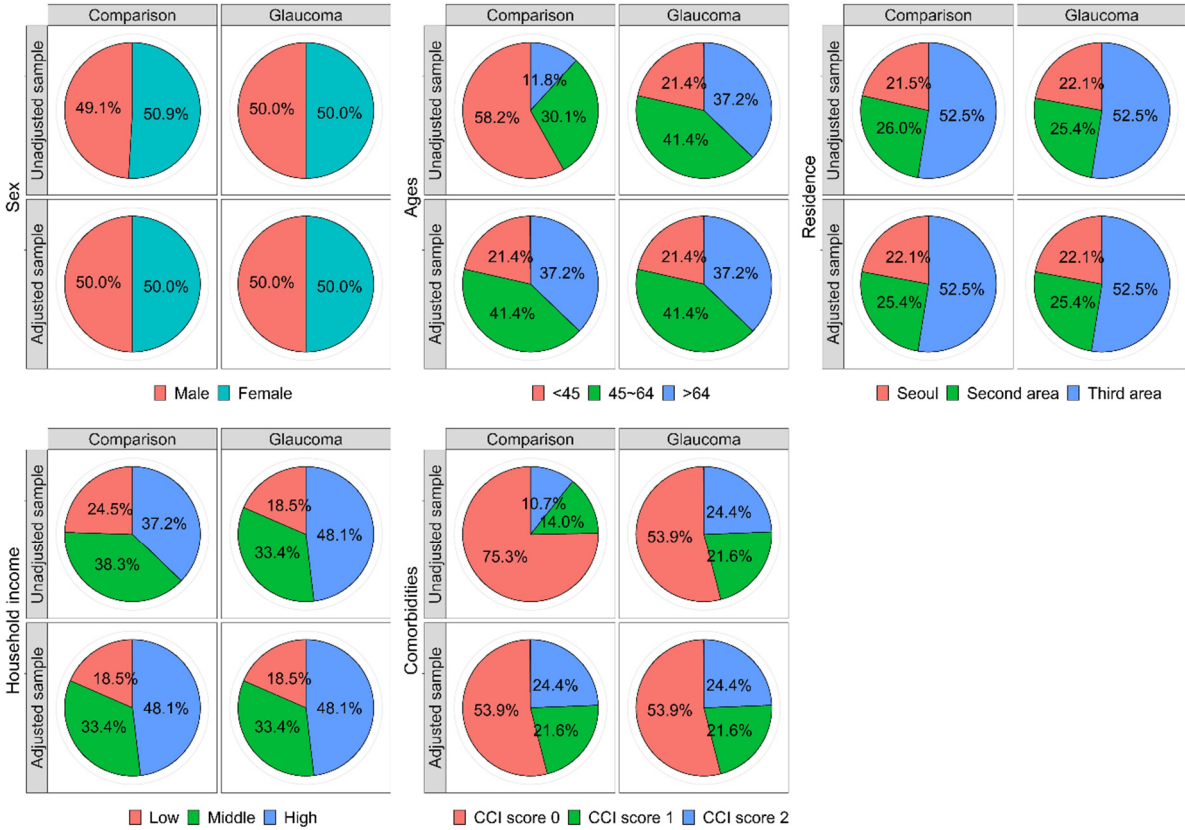

Figure S1. Balance plot for 5 variables before and after matching.

Supplement: Supplementary file 1 [file diagnostics-12-02992-s001.zip › diagnostics-2017043-Figure S1.pdf]
